# Enhanced recovery after minimally invasive valve surgery – ultra-fast extubation versus standard weaning strategy

**DOI:** 10.1038/s41598-025-21830-9

**Published:** 2025-10-08

**Authors:** Jacqueline Kruse, Isabel Krasny, Marwan Hamiko, Ömür Akhavuz, Eissa Alaj, Christian Bode, Marc Rohner, Ali El-Sayed Ahmad, Florian Piekarski, Mark Coburn, Markus Velten, Maria Wittmann, Enzo Lüsebrink, Farhad Bakhtiary, Miriam Silaschi

**Affiliations:** 1 Department of Cardiac Surgery, University Hospital Bonn, 53127 Bonn, Germany; 2 Department of Anesthesiology and Operative Intensive Care Medicine, University Hospital Bonn, 53127 Bonn, Germany; 3 Department of Anesthesiology and Pain Management, Division of Cardiovascular and Thoracic Anesthesiology, University of Texas Southwestern Medical Center, Dallas, TX 75390 USA; 4 Heart Center Bonn, Department of Medicine II, University Hospital Bonn, 53127 Bonn, Germany

**Keywords:** Ultra-fast extubation, Minimally invasive valve surgery, Weaning strategy, Medical research, Outcomes research

## Abstract

Immediate extubation in the operating room (OR) following cardiac surgery remains controversial. In 2021, our institution implemented a fast-track protocol that incorporated ultra-fast extubation (ORE) for minimally invasive heart valve surgery. We retrospectively evaluated outcomes and cost-effectiveness in comparison to standard extubation in the ICU (ICE). We retrospectively reviewed 615 minimally invasive valve surgeries performed between 2019 and 2023. Among these, 228 patients were extubated in the ICU, if possible, within 6 h as per current guidelines, while 387 patients underwent immediate ultra-fast extubation in the operating room (ORE) at the end of the surgery. Baseline variables were compared, and case-control matching was performed based on significantly different factors: Age, BMI and cross clamp times, resulting in two matched groups of 122 each. After matching, baseline variables were comparable between groups. Patients who underwent ORE had shorter ICU and hospital stays, were less likely to require ventilation for more than 24 h, and had significantly less often respiratory failure. Rates of repeat intubation did not differ significantly between groups. In multivariate logistic regression analysis, ORE, aortic cross clamp time, and EuroSCORE II were independent predictors of prolonged ventilation (> 24 h). However, conversion to sternotomy, previous cardiac surgery, gender, and history of smoking were not significantly associated with prolonged ventilation. ORE also led to reduced costs (Standard mean difference: 10 247€, *p* = 0.03). Ultra-fast extubation after minimally invasive valve surgery was associated with improved respiratory outcomes and lower costs compared to standard extubation in the ICU, with no increase in re-intubation rates. These findings support the clinical feasibility and safety of OR extubation in selected patients at experienced centers.

## Introduction

Ultra-fast extubation in cardiac surgery remains a topic of ongoing debate. Despite advances in minimally invasive techniques, characterized by smaller incisions and reduced surgical trauma, immediate postoperative extubation continues to be controversial^1^.

The concept of Enhanced Recovery After Surgery (ERAS) was introduced in the late 1990s for general surgical procedures and later adapted for cardiac surgery^1^. Ultra-fast extubation represents a core component of ERAS protocols, which aim to enhance clinical outcomes and shorten recovery time through a multidisciplinary, recovery-oriented approach. Over time, ERAS protocols have evolved and shown efficacy in reducing both ICU and overall hospital length of stay^1^. Additionally, they have been associated with decreased transfusion requirements, lower healthcare costs, and improved quality of life, particularly in gastrointestinal surgery^2^.

Nevertheless, concerns regarding potential failures of fast-track protocols persist and merit careful evaluation. In 2021, our center implemented a major revision to postoperative care by introducing routine ultra-fast extubation in the OR immediately following elective minimally invasive heart valve procedures.

This retrospective analysis aimed to assess the safety, clinical outcomes, and cost-effectiveness of OR extubation (ORE) compared to a matched cohort of patients extubated in the ICU, usually within six hours post-operatively, as recommended by current guidelines.

## Materials and methods

### Study design and patient population

This was a retrospective, single-center study. We performed minimally invasive heart valve surgery between January 2019 and July 2023 in 615 patients. Of these, 228 patients were extubated in the intensive care unit (“ICE”), while 387 patients underwent ultra-fast extubation in the operating room (“ORE”). We compared baseline variables and performed case-control matching based on three significantly different variables: age, BMI, and cross-clamp times. Matching resulted in two groups of 122 patients each: the ORE and ICE groups.

Matching was not performed according to EuroSCORE II as it was not statistically significant between both groups and as operative risk according to EuroSCORE II does not affect extubation strategy per se. In the resulting matched groups there is a tendency towards a higher EuroSCORE II in the ICU cohort but this was rather numerical (0.2% difference) than clinically relevant.

### Surgical technique

At the time of skin incision, patients are systemically heparinized to achieve an activated clotting time (ACT) > 450 s. Extracorporeal circulation (ECC) is established percutaneously via the femoral vessels, or alternatively via the axillary artery through a small cut-down, depending on aortic calcification or other vascular anomalies identified on preoperative full-body CT imaging.

The procedure is typically performed under normothermia, although mild hypothermia may be applied as clinically indicated. Venous cannulation includes an additional jugular cannula in patients weighing over 80 kg or undergoing tricuspid valve surgery^3^. A right thoracotomy is performed via the second or third intercostal space (approximately 6 cm incision), with the ribs accessed during apnea. A soft tissue retractor is used, and a 3D endoscope is introduced above the thoracotomy site. CO_2_ insufflation is applied through the endoscopic port. The pericardium is opened above the phrenic nerve.A transthoracic aortic clamp is inserted through a separate stab incision. Vents are placed in the ascending aorta and, if necessary, in the right upper pulmonary vein for aortic valve surgery. Cristalloid cardioplegia (1500–2000 mL) is administered via the aortic root or directly into the coronary ostia in cases of significant aortic regurgitation.

For aortic valve procedures, following adequate cardioplegia, the aorta is opened, and the valve is repaired or replaced. The coronary ostia are inspected, and the aorta is closed using a double-layer continuous suture technique.

In mitral valve surgery, a retraction suture is placed on the right atrium to expose the interatrial groove, and the oblique sinus is mobilized. The left atrium is opened, and the mitral valve is either repaired or replaced. Closure is performed with continuous double-layer suturing.

Before releasing the aortic cross-clamp, a temporary pacing wire is positioned in the right ventricle. De-airing is achieved by recruiting ventilation and briefly reducing venous return. Once reperfusion is sufficient, ventilation is resumed, and cardiac rhythm is stabilized. ECC is gradually weaned, and the venous cannula is removed with manual compression. The femoral arterial line is closed using a percutaneous vascular closure device.

Protamine is administered at a 1:1 ratio to reverse heparinization. Additionally, tranexamic acid (5 g total) is given—beginning with a 1 g bolus followed by continuous infusion over 6 h. Surgical sites (pleura, pericardium, and incision) are then inspected for hemostasis.

In 2021, the Department of Anesthesiology and Surgical Intensive Care Medicine implemented a standardized intraoperative management protocol, including ultra-fast-track extubation for patients undergoing minimally invasive cardiac surgery (MICS), in accordance with Enhanced Recovery After Surgery (ERAS) principles^3^. Since then, this approach has become the standard of care in our department.

*Anesthesia induction and maintenance*: All patients received standardized general anesthesia. Following preoxygenation, anesthesia was induced using a combination of propofol, remifentanil or sufentanil, and rocuronium. Airway management was performed with a single-lumen endotracheal tube in all cases, as one-lung ventilation (OLV) was not required. Video laryngoscopy was used based on individual airway assessment. Maintenance of anesthesia was achieved with volatile anesthetics during CPB, unless contraindications such as malignant hyperthermia were suspected. All intraoperative ventilation followed lung-protective protocols, in accordance with ERACS guidelines, which emphasize low tidal volumes and PEEP to reduce postoperative pulmonary complications^4^.

*Monitoring*: Standard intraoperative monitoring included five-lead ECG with ST segment analysis, invasive arterial pressure via ultrasound-guided left radial artery catheterization, central venous pressure, cerebral oximetry (NIRS), end-tidal CO_2_, bladder core temperature, bispectral index (BIS), and continuous transesophageal echocardiography (TEE). Patients at risk for pulmonary hypertension or right ventricular dysfunction were monitored using a pulmonary artery catheter.

*Temperature management*: Temperature management was integral to the enhanced recovery strategy. Active warming was initiated pre-induction using heating blankets and warming mats, with infusion warming devices used throughout. In on-pump procedures, normothermia was maintained via CPB. Body temperature was continuously monitored via bladder catheterization.

*Pain management*: We employed a multimodal pain regimen including intraoperative chest wall infiltration and postoperative non-opioid analgesics, consistent with ERACS recommendations to minimize opioid use and support fast-track recovery. Chest wall infiltration with 0.2% ropivacaine (10 mL) was performed by the surgeon for additional analgesia. Postoperatively, a multimodal analgesic regimen was applied using intravenous metamizole or paracetamol, with piritramide as needed. Regional anesthesia techniques (e.g., PECS blocks) were not routinely used.

**Fig. 1:**
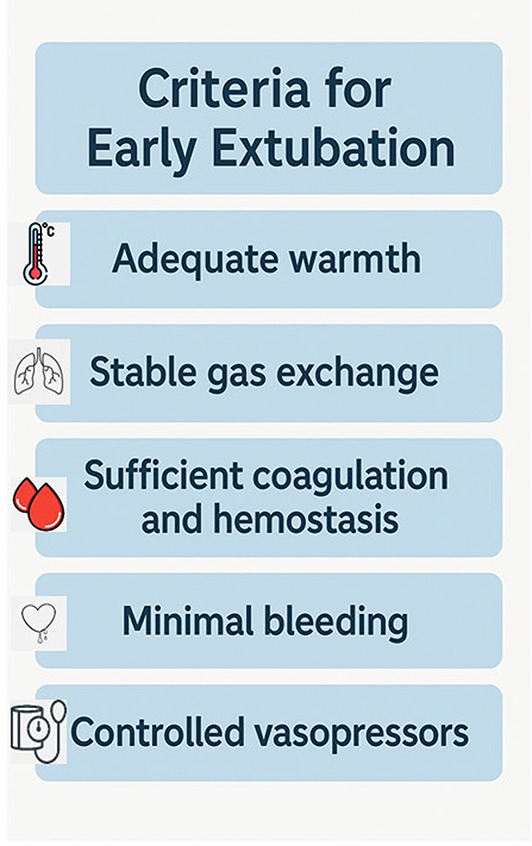
Criteria for early extubation.

*Ultra-fast extubation in the operating room*: ORE is performed for MICS procedures as part of the Enhanced Recovery After Surgery (ERAS) protocol. Criteria for extubation include adequate warmth, stable gas exchange, sufficient coagulation and hemostasis, minimal bleeding, and controlled vasopressors (Fig. 1).

### Costs analysis

The costs were calculated based on the hospital’s official billing records. In Germany, billing is standardized for all procedures, including any additional interventions and materials, according to the “German Medical Fee Schedule” and the Operating Procedures Catalogue. Since billing practices are uniform across institutions, the data from our hospital are representative and can be generalized.

### Statistical analysis

Data were collected and analyzed retrospectively. Categorical variables are presented as absolute numbers and percentages, with the denominator specified for each value. Continuous variables are reported as mean values with 95% confidence intervals (lower and upper) unless stated otherwise. A post-hoc power analysis for the primary endpoint (prolonged mechanical ventilation > 24 h) with 122 patients per group, an alpha of 0.05 and an observed effect size based on event rates (0% vs. 18.9%) showed a power of > 99.9%, confirming the adequacy of the sample size.

A 1:1 case-control matching based on age, BMI, and cross-clamp times was conducted to create more comparable cohorts of ORE and ICE for sensitivity analysis. The 1:1 matching was performed automatically using MedCalc software (version 20.218), which employs an automated matching algorithm within the specified caliper distances, which can be manually adjusted. No replacements were made. Matching and statistical analyses were carried out using MedCalc software (MedCalc Software Ltd., Ostend, Belgium). MedCalc uses a greedy matching algorithm within specified caliper distances. In interations the total standardized bias is minimized. The matching ratio is 1 on 1, without replacement. A paired *t*-test is used to assess comparability of baseline characteristics between matched groups^6^.

## Results

### Baseline characteristics

Before matching, BMI, creatinine and aortic cross clamp times were significantly different between the cohorts (see Table 1). After matching, groups had nearly identical ages (ORE: 63.51 years vs. ICE: 63.71 years, *p* = 0.87), with a more comparable sex distribution (ORE: 31.5% vs. ICE: 34.0%, *p* = 0.32). There was no significant difference in BMI between the two groups after matching (ORE: 26.7 vs. ICE: 26.6, *p* = 0.99). The Logistic EuroSCORE II was numerically lower in the ORE group (1.2%) compared to the ICE group (1.4%), though the difference was not statistically significant (*p* = 0.08). LVEF was also similar between the groups after matching (ORE: 58.3% vs. ICE: 57.8%, *p* = 0.31), and creatinine levels were nearly identical (ORE: 0.91 mg/dl vs. ICE: 0.95 mg/dl, *p* = 0.07). The prevalence of COPD was higher in the ORE group (24.5%) compared to the ICE group (12.2%), though this difference was also not statistically significant (*p* = 0.12). An increase in ORE was also seen after the in-house ERAS protocol was developed in 2021 (before 2021: 9.82% vs. after 2021: 73.16%; *p* = 0.26). ECMO therapy was used in 2.60% overall (16/615). Other comorbidities, such as coronary artery disease, diabetes, hypertension, and previous strokes, were similar between the two groups after matching. Baseline variables of the overall and matched cohort can be seen in Table 1.

**Table 1:** Baseline variables before and after matching ORE and ICE.

Baseline parameters	Before matching			After matching		
	ORE (n = 387)	ICE (n = 228)	*p*-value	ORE (n = 122)	ICE (n = 122)	*p*-value
Age, years	61.93 (60.7–63.1)	62.8 (61.2–64.3)	0.36	63.51 (61.9–65.1)	63.71 (62.1–65.4)	0.87
Sex (male), n (%)	232 (59.9)	150 (65.8)	0.15	77 (31.5)	83 (34.0)	0.32
BMI, kg/m^2^	25.8 (25.3–26.3)	27.1 (26.4–27.8)	**0.04**	26.7 (25.9–27.3)	26.6 (25.9–27.5)	0.99
Logistic EuroSCORE II, %	1.3 (1.2–1.4)	1.5 (1.3–1.7)	0.18	1.2 (1.0–1.3)	1.4 (1.2–1.6)	0.08
LVEF, %	59.1 (58.3–60.0)	58.3 (57.2–59.5)	0.28	58.3 (56.8–59.8)	57.8 (56.1–59.4)	0.31
Glomerular filtration rate, ml/min	81.5 (79.6–83.5)	79.9 (77.2–82.5)	0.34	81.6 (78.1–84.9)	81.4 (77.9–84.8)	0.93
Previous Cardiac Surgery, n (%)	18 (4.6)	10 (4.4)	0.88	2 (1.6)	6 (4.9)	0.14
COPD, n (%)	15 (3.9)	14 (6.1)	0.20	12 (24.5)	6 (12.2)	0.12
BSA (m^2^)	1.9 (1.9–1.9)	2.3 (1.6–2.9)	0.17	1.95 (1.9–2.0)	1.97 (1.9–2.0)	0.71
Coronary artery disease, n (%)	93 (24.0)	51 (22.5)	0.90	34 (27.9)	29 (23.8)	0.90
Previous PCI, n (%)	226/385 (58.7)	127/228 (55.7)	0.47	73 (59.8)	66 (54.1)	0.41
Permanent Pacemaker/ internal defibrillator present, n (%)	18 (4.6)	11 (4.8)	0.92	6 (4.9)	5 (4.1)	0.78
Creatinine, mg/dl	0.93 (0.9–1.0)	1.0 (0.9–1.0)	**0.01**	0.91 (0.9–1.0)	0.95 (0.9–1.0)	0.07
History of smoking, n (%)	100 (25.8)	55 (24.1)	0.79	38 (31.1)	35 (28.6)	0.68
Packyears	4.02 (2.8–5.2)	3.9 (2.2–5.6)	0.91	4.33 (1.9–6.8)	4.21 (1.9–6.4)	0.94
Diabetes melitus, n (%)	38 (9.8)	22 (9.6)	0.74	16 (13.1)	13 (10.7)	0.59
History of previous stroke, n (%)	13 (3.4)	12 (5.3)	0.25	5 (4.1)	6 (4.9)	0.74
Arterial hypertension, n (%)	221 (57.1)	139 (60.9)	0.29	72 (59)	74 (60.7)	0.89
Peripheral arterial disease, n (%)	11 (2.8)	6 (2.6)	0.88	5 (4.1)	3 (2.5)	0.49
Carotid stenosis > 50%, n (%)	6 (1.6)	1 (0.26)	0.21	1 (0.82)	0 (0)	0.31
NYHA, n (%)						
I	97 (25.0)	49 (21.5)	0.16	22 (18.0)	26 (21.3)	0.87
II	184 (47.6)	95 (41.7)		59 (48.4)	54 (44.3)	
III	93 (24.1)	73 (32.0)		35 (28.7)	35 (28.7)	
IV	13 (3.3)	11 (4.8)		6 (4.9)	7 (5.7)	

### Operative characteristics and outcomes

Before matching, the ORE group had shorter cross-clamp times (81.97 vs. 133.3 min, *p* < 0.01) and required fewer packed red blood cells (1.53 vs. 3.36 units, *p* < 0.01) and fresh frozen plasma transfusion (0.61 vs. 1.52 units, *p* < 0.01) compared to the ICE group. In terms of valve procedures, the ORE group had a significantly lower percentage of mitral valve procedures (62.0% vs. 77.1%, *p* < 0.01) but a higher percentage of aortic valve procedures (30.2% vs. 19.4%, *p* = 0.03). The rate of complications was lower in the ORE group, with significantly fewer cases of pneumonia (3.6% vs. 9.7%, *p* < 0.01) and superficial wound infections (1.8% vs. 4.8%, *p* = 0.03). However, the ORE group had a higher incidence of renal failure requiring dialysis (3.3% vs. 1.2%, *p* < 0.01). Additionally, the ORE group had a shorter ICU stay (2.71 days vs. 6.82 days, *p* < 0.01) and hospital stay (12.01 days vs. 17.43 days, *p* < 0.01). Numpber of pRBC transfusion was not associated with prolonged ventilation > 24 h in logistic regression analysis of the overall cohort (OR 1.01 [95% CI 0.9–1.0], *p* = 0.18).

**Fig. 2:**
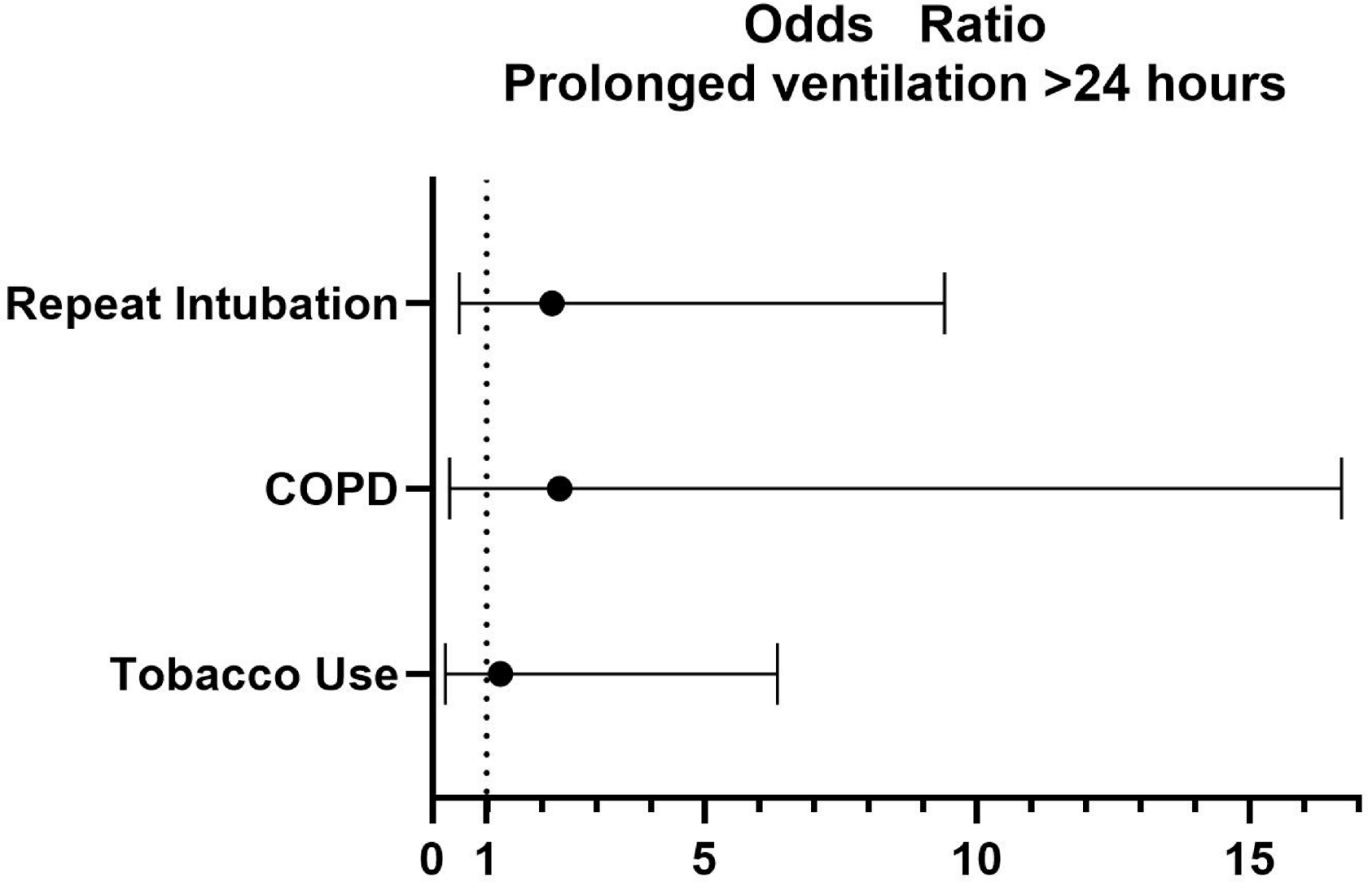
Odds ratios for prolonged ventilation (> 24 h).

After matching, both groups showed similar aortic cross-clamp times (94.23 vs. 94.84 min, *p* = 0.92) and valve procedure rates (e.g., 72.1% vs. 61.5% for mitral valve procedures, *p* = 0.10; 22.9% vs. 32.8% for aortic valve procedures, *p* = 0.11). Blood product use remained significantly lower in the ORE group (1.37 vs. 3.54 units of pRBC, *p* < 0.01; 0.51 vs. 1.33 units of FFP, *p* < 0.01). Complications including pneumonia (4.0% vs. 6.6%, *p* = 0.37) and superficial wound infections (0% vs. 3.3%, *p* = 0.12) did not differ significantly. The ORE group had numerically shorter ICU stay (2.54 days vs. 7.1 days, *p* = 0.07) and hospital stay (11.9 days vs. 16.9 days, *p* = 0.11), though neither difference was statistically significant. Despite early extubation of ORE patients, rate of repeat intubation was not significantly different. Respiratory failure and prolonged ventilation were more frequent in ICE patients (see Fig. 2).

Further results are listed in Table 2.

**Table 2:** Procedural data and 30 days results, before and after matching ORE versus ICE.

Procedural data and 30-day results	Before matching			After matching		
	ORE (n = 387)	ICE (n = 227)	*p*-value	ORE (n = 122)	ICE (n = 122)	*p*-value
Cross Clamp time, minutes	81.97 (78.9–85.0)	133.3 (124.9-141.7)	**< 0.01**	94.23 (88.1–100.0)	94.84 (88.4-101.2)	0.92
Mitral valve procedure, n (%)	240 (62.0)	175 (77.1)	**< 0.01**	88 (72.1)	75 (61.5)	0.10
Aortic valve procedure, n (%)	117 (30.2)	44 (19.4)	**0.03**	28 (22.9)	40 (32.8)	0.11
Tricuspid valve procedure, n (%)	7 (1.8)	3 (1.3)	0.75	2 (1.6)	0 (0)	0.49
Mitral and aortic valve, n (%)	9 (2.3)	1 (0.4)	0.10	2 (1.6)	2 (1.6)	1.00
Mitral and tricuspid valve, n (%)	14 (3.6)	5 (2.2)	0.47	2 (1.6)	5 (4.1)	1.00
pRBC, n (%)	1.53 (1.2–1.8)	3.36 (1.9–4.8)	0.14	1.37 (0.98–1.8)	3.54 (1.1–5.9)	**< 0.01**
FFPs, n (%)	0.61 (0.4–0.8)	1.52 (1.1-2.0)	**< 0.01**	0.51 (0.2–0.8)	1.33 (0.8–1.9)	**< 0.01**
Conversion to sternotomy, n (%)	2 (0.51)	5 (2.2)	0.06	1 (0.8)	1 (0.8)	1.00
ASD repair, n (%)	31 (8.0)	24 (10.6)	0.29	9 (7.4)	11 (9.0)	0.61
Delirium, n (%)	29 (7.5)	30 (13.2)	**0.02**	11 (9.0)	16 (13.1)	0.40
30-day mortality, n (%)	1 (0.3)	8 (3.5)	**< 0.01**	0 (0)	5 (4.0)	**0.02**
TIA, n (%)	1 (0.3)	1 (0.4)	0.70	1 (0.8)	1 (0.8)	0.99
Stroke, n (%)	5 (1.3)	4 (1.8)	0.64	3 (2.5)	4 (3.3)	0.68
All cause in hospital mortality, n (%)	2 (0.5)	14 (6.1)	**< 0.01**	0 (0)	6 (4.9)	**0.04**
Aphasia, n (%)	2 (0.5)	2 (0.8)	0.59	2 (1.6)	2 (1.6)	1.00
Aspiration, n (%)	3 (0.7)	2 (0.8)	0.89	1 (0.8)	0 (0)	0.32
Atrial fibrillation, n (%)	105 (27.1)	68 (29.9)	0.58	38 (31.1)	35 (28.7)	0.74
Pericardial effusion, n (%)	28 (7.2)	12 (5.3)	0.36	5 (4.0)	9 (7.4)	0.45
Cardiogenic shock, n (%)	6 (1.5)	2 (0.6)	0.48	1 (0.8)	2 (1.6)	0.55
ECMO, n (%)	1 (0.2)	15 (6.6)	0.75	1 (0.8)	6 (4.9)	0.11
GI bleeding, n (%)	1 (0.3)	3 (1.3)	0.11	0 (0)	2 (1.6)	0.49
GI ischemia, n (%)	0 (0)	4 (1.7)	**< 0.01**	0 (0)	3 (2.5)	0.08
Hemiplegia, n (%)	2 (0.5)	7( 3.1)	0.75	1 (0.8)	5 (4.0)	0.23
Inotropic support for 24 h	57 (14.7)	53 (23.3)	**0.01**	20 (16.4)	29 (23.8)	0.13
LCOS, n (%)	5 (1.3)	15 (6.6)	**< 0.01**	1 (0.8)	8 (6.6)	**0.03**
Left ventricular decompensation, n (%)	37 (9.5)	29 (12.8)	0.20	14 (11.5)	11 (9.0)	0.78
Multi-organ failure, n (%)	2 (0.5)	6 (2.6)	**0.02**	0 (0)	4 (3.3)	0.12
Need for pacemaker, n (%)	16 (4.1)	10 (4.4)	0.87	5 (4.0)	8 (6.6)	0.37
Pneumonia, n (%)	14 (3.6)	22 (9.7)	**0.005**	5 (4.0)	8 (6.6)	0.37
Prolonged ICU stay (≥ 3d), n (%)	56 (14.5)	71 (31.3)	**< 0.01**	12 (9.8)	37 (30.3)	**< 0.01**
Prolonged ventilation (≥ 24 h), n (%)	2 (0.5)	46 (20.3)	**< 0.01**	0 (0)	23 (18.9)	**< 0.01**
Repeated intubation, n (%)	23 (5.9)	26 (11.5)	**0.02**	7 (5.7)	12 (9.8)	0.27
Respiratory insufficiency, n (%)	39 (10.0)	59 (26.0)	**< 0.01**	13 (10.7)	29 (23.8)	**0.01**
Tamponade and re-exploration, n (%)	8 (2.0)	5 (2.2)	0.91	0 (0)	4 (3.3)	0.12
Superficial wound infection, n (%)	7 (1.8)	11 (4.8)	**0.03**	0 (0)	4 (3.3)	0.12
Renal failure, n (%)	13 (3.3)	23 (18.9)	**< 0.01**	3 (2.5)	12 (9.8)	**0.01**
Renal failure requiring dialysis, n (%)	5 (1.2)	14 (6.1)	**< 0.01**	3 (2.5)	7 (5.7)	0.19
ICU stay, days	2.71 (2.1–3.2)	6.82 (3.9–9.7)	**< 0.01**	2.54 (1.8–3.3)	7.1 (2.0-12.2)	0.07
Hospital stay, days	12.01 (11.1–12.9)	17.43 (13.8–21.0)	**< 0.01**	11.9 (10.7–13.3)	16.9 (10.8–23.2)	0.11
Coma, n (%)	1 (0.3)	2 (0.8)	0.56	0 (0)	1 (0.8)	0.31
Chronic renal failure, n (%)	21 (9.2)	16 (7.0)	**0.03**	3 (2.5)	6 (4.9)	0.45
Death, n (%)	1 (0.3)	8 (3.5)	**< 0.01**	0 (0)	7 (5.7)	**0.02**

Significant values are in bold.

Analysis of risk factors for prolonged ventilation (≥ 24 h) was performed through multivariate logistic regression analysis. In multivariate logistic regression analysis, extubation in the operating room was strongly associated with a lower likelihood of prolonged ventilation (OR: 0.02 (0.001-0.3)). Another significant predictor was EuroSCORE II (Odds ratio: 2.6 (1.1–6.3) although the wide confidence intervals indicate uncertainty.

**Fig. 3:**
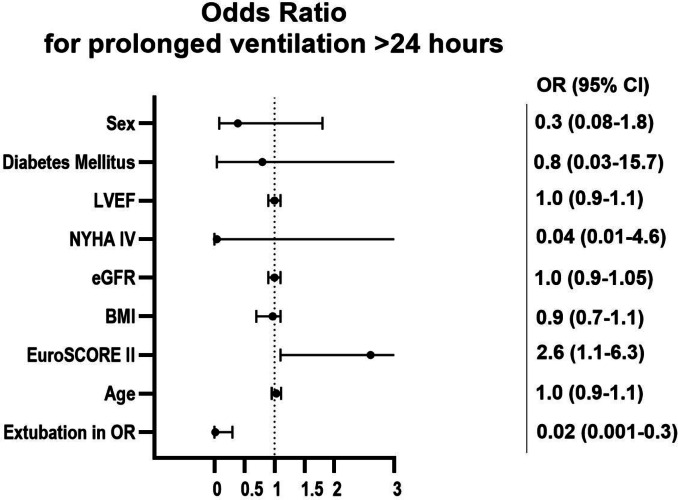
Odds ratio for predictors of prolonged ventilation (> 24 h).

Factors such as age (OR 1.0 (0.9–1.1)), previous cardiac surgery (OR: 4.5 (0.3–68.3) and COPD 2.9 (0.2–39.1)), BMI (OR 0.9 (0.7–1.1)), gender (OR 0.3 (0.08–1.8)), diabetes mellitus (OR 0.8 [0.03–15.7]), LVEF (OR 1.0 [0.9-1. 1]), NYHA-IV classification (OR 0.04 [0.01–4.6]) and eGFR (OR 1.0 [0.9–1.05]) did not show a significant influence. (See Figs. 2 and 3).

### Subanalysis for the reasons of re-intubation

Among the 49 patients who required re-intubation, the most common causes identified were re-exploration for bleeding into the pleural cavity (30.61%, 15/49) and pneumonia (16.32%, 8/49). Notably, among those with pneumonia, aspiration was documented in 37.5% (3/8) of the cases. Re-intubation due to re-exploration for cardiac tamponade was observed in 10.20% (5/49). 36.73% (18/49) were re-intubated because of general respiratory failurenspecified cause (respiratory failure).

The rate of re-intubation after matching was 7.8% (19/244). ORE 5.7% (7/122) versus ICE 9.8% (12/122).

### Subanalysis for matching according to EuroScore II

In addition to the first matching we performed an additional subanalysis with matching according to EuroSCORE II. The same Matching algorithm was used with a maximum allowable difference of EuroSCORE II of 0.2%. This yielded 90 versus 146 patients with a mean EuroSCORE II of 1.29 ± 1.04% versus 1.21 ± 1.03% and confirmed our previous results: the two patient groups, ICE (n = 90) and ORE (n = 146), had comparable baseline variables regarding age, gender, BMI, LVEF and prevalence of smoking and diabetes (see Table 3).

**Table 3:** Matching according to EuroScore II.

	ICE (*n* = 90)	ORE (*n* = 146)	*p*-value
Age (years), CI	63.69 (61.36–66.02)	62.72 (60.86–64.58)	0.52
Male, n (%)	58 (64.44)	96 (65.75)	0.84
BMI (kg/m^2^), CI	26.47 (25.51–27.44)	26.04 (25.26–26.83)	0.50
History of smoking, n (%)	20 (22.22)	32 (21.91)	0.94
Diabetes mellitus, n (%)	6 (6.67)	18 (12.33)	0.33
COPD, n (%)	8 (8.89)	5 (3.42)	0.07
Chronic renal failure, n (%)	3 (3.33)	8 (5.48)	0.22
NYHA classification, n (%)			
I	14 (15.56)	32 (21.91)	0.38
II	41 (45.56)	72 (49.32)	
III	31 (34.44)	37 (25.34)	
IV	4 (4.44)	5 (3.42)	
LVEF (%), CI	58.36 (56.51–60.21)	58.78 (57.43–60.14)	0.71
Crossclamping time (min.), CI	**146.64 (133.35-159.93)**	**83.21 (78.13–88.28)**	**< 0.01**
Prolonged ventilation (> 24 h), n (%)	**13 (14.44)**	**0**	**< 0.01**
Prolonged ICU stay (> 3d), n (%)	**22 (24.44)**	**15 (30.82)**	**0.01**
ECMO, n (%)	**3 (3.33)**	**0**	**0.03**
Pneumonia, n (%)	**8 (8.89)**	**1 (0.68)**	**< 0.01**
Aspiration, n (%)	1 (1.11)	0	0.20
Respiratory insufficiency, n (%)	16 (17.78)	14 (9.59)	0.06
Repeated intubation, n (%)	11 (12.22)	10 (6.85)	0.22
Deep wound infection, n (%)	4 (4.44)	1 (0.68)	0.05
MOFS, n (%)	2 (2.22)	0	0.07
Hospital stay (days), CI	**14.37 (12.32–16.43)**	**11.11 (10.16–12.06)**	**< 0.01**
Death, n (%)	2 (2.22)	0	0.07
30 day mortality, n (%)	2 (2.22)	0	0.07

Significant values are in bold.

The duration of cardiovascular surgery (crossclamping time) was significantly longer in the ICE group compared to the ORE group 146 versus 83 min; *p* < 0.01)

In terms of postoperative outcomes, the ICE group exhibited prolonged ventilation requirements more often (> 24 h), also incidence of pneumonia was higher and intensive care stays were longer. The average length of hospitalization is also significantly longer for ICE (around 14.4 days) compared to ORE (around 11 days), *p* < 0.01.

### Subanalysis of patient characteristics and outcome of pre- and post-ERAS era

The study included 615 patients, with 124 in the pre-ERAS group and 491 in the post-ERAS group. Baseline characteristics, including age, sex, BMI, comorbidities, EuroSCORE II, NYHA classification, and LVEF, showed no significant differences between groups.

Following ERAS implementation, several perioperative and postoperative outcomes differed significantly. Ultra-fast extubation increased from 16.94 to 74.54% (*p* < 0.01), while prolonged ventilation (> 24 h) decreased from 15.32 to 5.70% (*p* < 0.01). Prolonged ICU stay (> 3 days) declined from 29.03 to 17.92% (*p* < 0.01). Hospital stay was shortened from 16.95 to 13.26 days (*p* = 0.04).

Postoperative respiratory events were also reduced: pneumonia (10.48% vs. 4.68%, *p* = 0.02) and respiratory insufficiency (25.81% vs. 13.24%, *p* < 0.01). Rates of aspiration and repeated intubation did not differ significantly between groups. For further Information see Table 4.

**Table 4:** Baseline characteristics and outcome of re- and post-ERAS era.

	Pre-ERAS (n = 124)	Post-ERAS (n = 491)	*p*-value
Age (years), CI	61.65 (59.23–64.06)	62.39 (61.40-63.39)	0.53
Male, n (%)	72 (58.06)	310 (63.14)	0.30
BMI (kg/m^2^), CI	25.69 (24.87–26.52)	26.46 (26.01–26.91)	0.13
History of smoking, n (%)	22 (17.74)	133 (27.09)	0.09
Diabetes mellitus, n (%)	8 (6.45)	52 (10.59)	0.36
COPD, n (%)	5 (4.03)	24 (4.89)	0.69
Chronic renal failure, n (%)	7 (5.65)	30 (6.11)	0.59
EuroSCORE II, n (%)	1.40 (1.16–1.64)	1.39 (1.26–1.53)	0.96
NYHA classification, n (%)			
I	28 (22.58)	118 (38.29)	0.27
II	48 (38.71)	231 (47.05)	
III	41 (3.23)	125 (25.46)	
IV	7 (5.65)	17 (3.46)	
LVEF (%), CI	59.30 (57.61-61.00)	58.75 (57.98–59.51)	0.53
Crossclamping time (min.), CI	**178.84 (168.88–188.80)**	**81.10 (78.81–83.38)**	**< 0.01**
Ultra-fast extubation (ORE), n (%)	**21 (16.94)**	**366 (74.54)**	**< 0.01**
Prolonged ventilation (> 24 h), n (%)	**19 (15.32)**	**28 (5.70)**	**< 0.01**
Prolonged ICU stay (> 3d), n (%)	**36 (29.03)**	**88 (17.92)**	**< 0.01**
ICU stay (days), CI	6.00 (3.77–8.23)	3.73 (2.41–5.04)	0.12
Hospital stay (days), CI	**16.95 (14.07–19.83)**	**13.26 (11.61–14.91)**	**0.04**
Pneumonia, n (%)	**13 (10.48)**	**23 (4.68)**	**0.02**
Aspiration, n (%)	2 (1.61)	3 (0.61)	0.27
Respiratory insufficiency, n (%)	**32 (25.81)**	**65 (13.24)**	**< 0.01**
Repeated intubation, n (%)	13 (10.48)	36 (7.33)	0.07
Deep wound infection, n (%)	**4 (3.23)**	**2 (0.41)**	**< 0.01**
ECMO, n (%)	**8 (6.45)**	**8 (1.63)**	**< 0.01**
Death, n (%)	3 (2.42)	9 (1.83)	0.81
30 day mortality, n (%)	3 (2.42)	7 (1.43)	0.54

Significant values are in bold.

### Subanalysis of patient characteristics post-ERAS era (ORE vs. ICE)

A subanalysis of patients within ERAS period (after 2021) identified characteristics of patients unsuitable for ultra-fast extubation. The results showed that after the introduction of the ERAS protocol, patients extubated on ICU had significantly higher BMI and more often chronic renal insufficiency resulting in significantly higher creatinine values (*p* < 0.01). See Table 5 for more information.

**Table 5:** Baseline characteristics of post-ERAS era for ORE versus ICE.

	ICE (n = 125)	ORE (n = 366)	*p*-value
Age (years), CI	63.28 (61.44–65.12)	62.09 (60.91–63.27)	0.31
Male, n(%)	90 (72)	220 (60.11)	0.02
History of smoking, n (%)	37 (29.60)	96 (26.23)	0.71
Diabetes Mellitus	16 (12.80)	36 (9.84)	0.50
COPD	9 (7.20)	15 (4.10)	0.17
Chronic renal failure	**11 (8.80)**	**19 (5.19)**	**< 0.01**
Creatinin level	**1.08 (0.92–1.24)**	**0.92 (0.89–0.94)**	**< 0.01**
Arterial hypertension	80 (64)	210 (57.38)	0.11
BMI (kg/m^2^)	**28.08 (27.03–29.12)**	**25.90 (25.43–26.38)**	**< 0.01**
EuroSCORE II	1.6 (1.2–1.9)	1.3 (1.2–1.5)	0.17
LVEF_%_	57.95 (56.42–59.48)	59.02 (58.14–59.90)	0.23
NYHA classification			
I	26 (20.80)	92 (25.14)	0.18
II	55 (44)	176 (48.10)	
III	36 (28.80)	89 (24.32)	
IV	8 (6.40)	9 (2.46)	

Significant values are in bold.

**Fig. 4:**
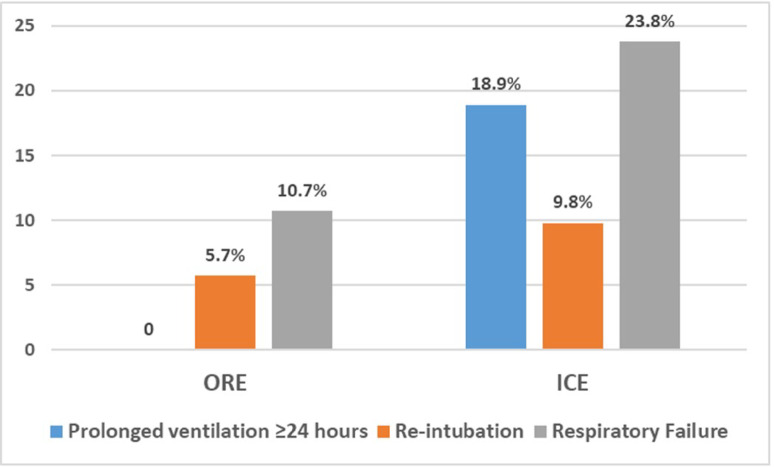
Rate of respiratory events in ORE versus ICU in matched cohorts.

Rate of respiratory events (prolonged ventilation ≥ 24 h, re-intubation, respiratory failure) in ORE versus ICU in matched cohorts are shown in Fig. 4.

The different length of cross-clamping time before and after the development of the ERAS-protocol is due to the use of automated technologies, such as titanium fasteners for knot tying and automated suturing devices, can significantly reduce operative times.

### Analysis of costs

**Fig. 5:**
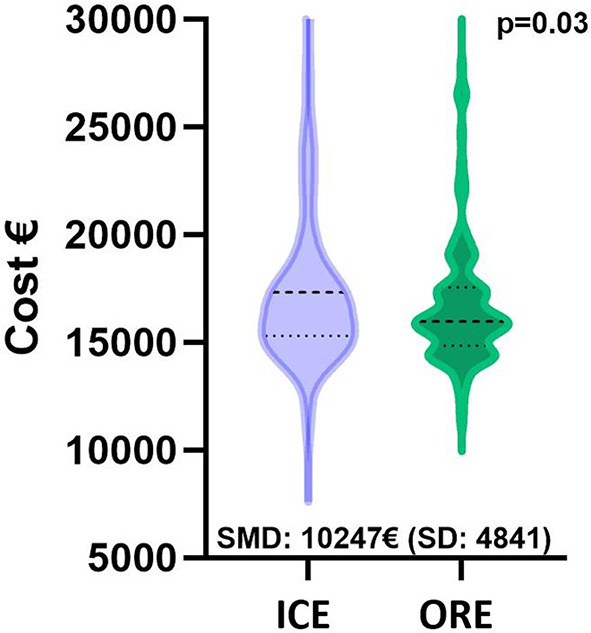
Violin plot of costs of the matched cohorts.

**Fig. 6:**
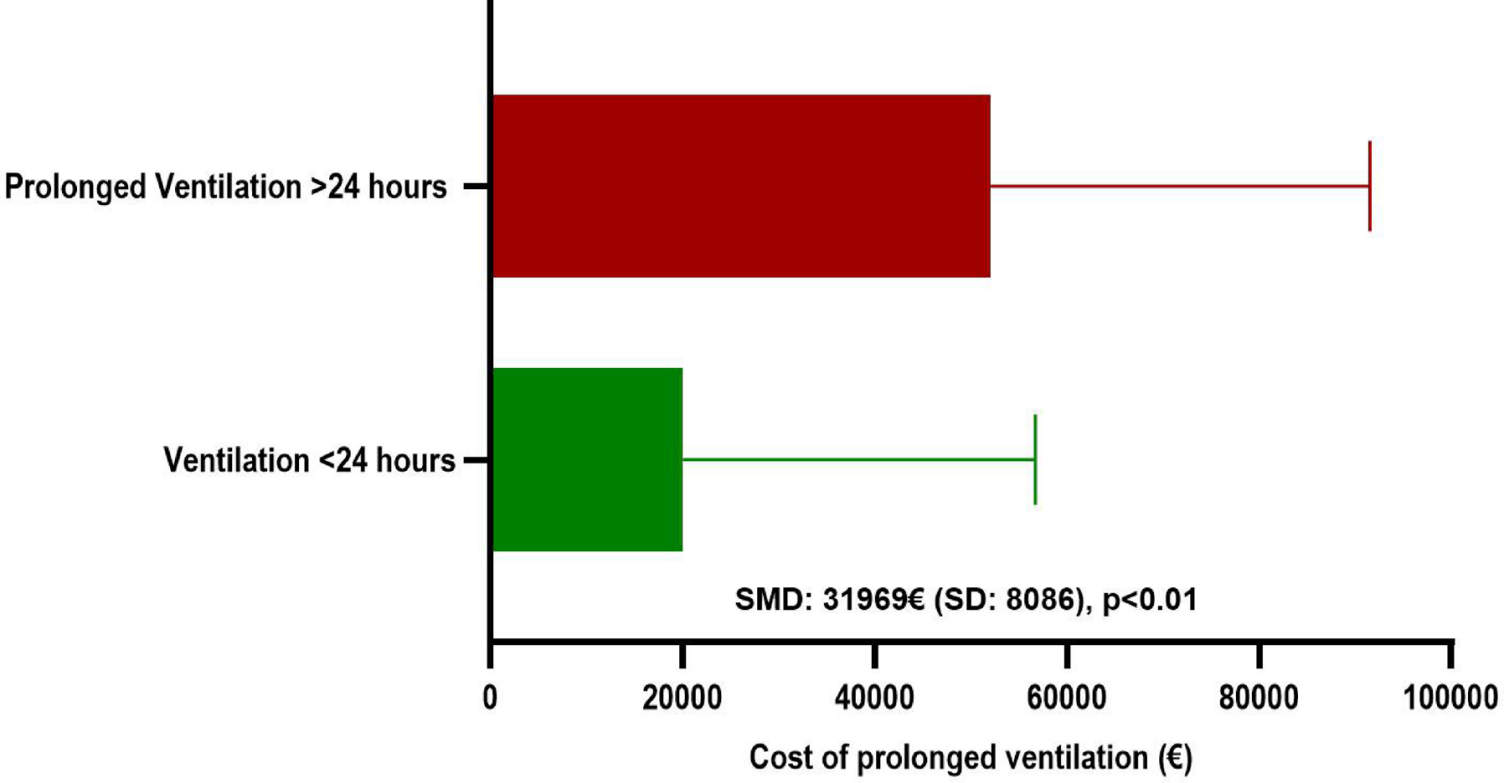
Diagram of the overall costs in patients with prolonged ventilation (> 24 h).

In a cost-efficiency analysis, ORE was associated with significant lower costs (*p* = 0.03) compared to ICE with a standard mean difference of 10,247 euros (Fig. 5). If the costs for the entire cohort are compared based on patients who underwent prolonged ventilation > 24 h and those who were ventilated < 24 h, difference increased (*p* < 0.01) with a SMD of 31,969 euros (Fig. 6).

## Discussion

This study shows that operating room extubation (ORE) in minimally invasive cardiac surgery is associated with a decreased risk of prolonged mechanical ventilation, without increasing complication or reintubation rates. A distinct shift in postoperative care strategy enabled a time-based comparison with reduced bias. To enhance comparability, case-control matching was performed, yielding two cohorts of 122 patients each. The unchanged reintubation rate despite ultra-fast extubation supports the safety of the ORE approach. Various subanalyses of our cohorts confirmed these results. While limitations exist, the findings suggest that ORE is a safe and non-inferior alternative to standard weaning protocols.

In terms of costs, the ORE group demonstrated a clear benefit, not only in the overall cohort but particularly when comparing patients ventilated < 24 h versus those ventilated ≥ 24 h.

These findings suggest that ultra-fast extubation may be associated with superior short-term results compared to the ICE approach. These results support the feasibility and positive impact of ORE in MIVS patients.

While other studies have not focused exclusively on ultra-fast extubation in the operating room, our findings align closely with those of centers investigating fast-track protocols with early extubation strategies (mostly within the first 6 h postoperatively)^5^. For example, Malvindi et al. assessed the impact of an ultra-fast track protocol on early extubation in minimally invasive transaxillary mitral valve surgery patients. The results showed that fast track extubation (within 6 h post-surgery) was linked to shorter Intensive Care Unit (ICU) stays and higher rates of discharge home within 7 days, compared to those who underwent non-fast track extubation^7^.

Among the fast track extubation group, patients who were extubated on the operating table had even shorter ICU stays (≤ 1 day) and were more likely to be discharged home by postoperative day 7. These results echo our findings, reinforcing the safety and efficacy of early extubation strategies in MIVS.

Previous studies have shown that early extubation, particularly ORE, is associated with shorter ICU and hospital stays, reduced resource utilization, and no increase in reintubation rates or postoperative complications. Randomized trials further link ORE to improved hemodynamic stability, fewer complications (e.g., pleural effusions), and reduced catecholamine use, supporting enhanced recovery^8–10^. These results align with our findings, which also demonstrate shorter ICU stays and fewer complications following ORE^11^.

Kiessling et al. reported fast-track (FT) failure associated with prolonged cardiopulmonary bypass (> 130 min) and total operative time (> 267 ± 74 min) in coronary artery bypass patients^12^. In our study, aortic cross-clamp time showed no significant impact, though the low number of reintubations limited statistical analysis. While Kiessling also found FT failure more frequent in patients with NYHA class III–IV, we did not observe this association in our cohort.

Similarly, Van Praet’s study also reported significant findings when comparing baseline characteristics. Specifically, chronic renal insufficiency and pre-existing coronary artery disease were associated with fast track failure, leading to prolonged ventilation times^13^.

Ge Y et al. compared the clinical outcomes of extubation in the operating room versus delayed extubation in patients over 60 years old undergoing minimally invasive mitral or aortic valve replacement surgery. It found that extubation in the operating room resulted in shorter surgery and recovery times, as well as reduced costs. While the study did not specify patient characteristics that would make operation room extubation unfeasible, it highlighted that longer surgical times, particularly prolonged aortic occlusion clamping, may reduce the likelihood of successful operation room extubation. However, specific contraindications for ultra-fast extubation were not detailed in the study^14^.

Certain patient populations with NYHA class III–IV, chronic renal insufficiency, or coronary artery disease, are at higher risk of fast-track failure and may be less suitable for ultra-fast extubation. In our matched cohorts, increased transfusion requirements in ICE patients suggest that individuals with impaired hemostasis or intraoperative bleeding were less likely to undergo ORE. Immediate extubation should be avoided in such cases; thus, ORE is not appropriate for all patients. However, following its success in minimally invasive valve surgery (MIVS), we have extended the protocol to select patients undergoing full sternotomy.

At our department, patients with a fragile aortic wall – noted during closure of aortotomy – regularly do not undergo ultra-fast extubation, as blood pressure peaks can occur more frequently in the recovery phase, increasing the risk of bleeding or aortic dissection. Older people are more frequently affected. However, they are then extubated within the first 6–8 h in the intensive care unit.

## Conclusion

Ultra-fast extubation is safe and cost-efficient and does not increase the risk of respiratory failure and reintubation. The relative risk of prolonged ventilation ≥ 24 h was significantly reduced in the ORE group, with a low re-intubation rate, however, our results might not universally apply to all hospitals since this study was conducted at a high volume center with routine ORE. Nevertheless, re-intubation rates were very low and given these findings, ORE strategy should not be discouraged in appropriate patients treated experienced heart valve centers following minimally invasive heart valve surgery.

Further studies are needed to validate these findings and investigate potential patient-specific factors that may influence the success of ORE in different populations.

### Limitations

The study is retrospective and includes all minimally invasive heart valve procedures from the period from 2019 to 2023. However, the concept of ultra-fast extubation was established at our department in 2021, which made comparison of contemporary to historic patients possible. A limitation of this study is that patients extubated within 6 h in the ICU could not be analyzed separately from those extubated within 24 h. The concept of ultra-fast extubation requires routine and this can only be achieved through experience, which may be achieved through a learning curve. Not only the anesthetists and anesthesia nurses need routine, but also the surgeons who perform the procedures.

Although we performed matching, bias cannot be excluded and conclusions can only be drawn very carefully. There is still a residual risk of bias in between the groups. Only a few variables were matched, as otherwise the groups would have been too small and the statistical significance would not have been clear.

Also, since cohorts were from two different time periods (before 2021 and after) a time-trend bias could still exsist.

## Data Availability

The datasets generated and analyzed during the current study are available from the corresponding author upon reasonable request. Due to ethical and privacy considerations, some data may be restricted, but will be made available to qualified researchers upon formal request.

## Abbreviations

ACT: Activated clotting time
ICU: Intensive care unit
NIRS: Near infrared spectroscopy monitoring
LVEF: Left ventricular ejection fraction
TEE: Trans-esophageal echocardiography
OR: Operating room
BIS: Bispectral index
MICS: Minimally invasive cardiac surgery
ORE: OR extubation
ICE: Intensive care extubation.
ERAS: Enhanced Recovery After Surgery
ECC: Extracorporeal circulation
COPD: Chronic obstructive pulmonary disease
NYHA: New York Health Association
BMI: Body mass index
MIVS: Minimally invasive valve surgery
CPBT: Cardio-pulmonary bypass time
FT: Fast track
DLT: Double-lumen tube
OLV: One lunge ventilation
